# Cerebral Hemorrhage

**Published:** 1902-10

**Authors:** Sydney A. Dunham

**Affiliations:** Buffalo, N.Y., 239 Delaware Avenue.


### ABSTRACT.

### Cerebral Hemorrhage.

By SYDNEY A. DUNHAM, M.D., Buffalo, N.Y.

*Report of Case Following Injury to Spine.*—Cerebral hemorrhage, properly speaking, means hemorrhage into substance of brain. If it follows an injury it may occur in any part of the encephalon. When into the meninges it is meningeal. When into the ventricle it is called ventricular. It is rare into the cortical substance, unless following traumatism. It may be directly under site of injury to the cranium or contrecoup.

I was present at autopsy one year ago where a young man was struck on right side of head, producing severe hemorrhage into substance of brain on left side. In the present case which I have reported the injury was on dorsal region of spine with no history of injury to the head, yet a hemorrhage was found into right cortical substance near the fissure of Rolando. In this case the hemorrhage was slow. Patient walked home, being a little dazed at the time. Slight hem-

Read before Buffalo Academy of Medicine, September 9, 1902.

iplegia of left side developing on the third day, when he sent for a physician. Left patella reflex exaggerated. Patient grew gradually worse until death, two months after injury.

There was no mental confusion, but drowsiness at times during first few weeks; no change in pupils, no disturbance in speech, no facial paralysis. The arms and legs would alternately be better and worse. The patient walked about the house the first week, ate well and slept well, expecting each day to be able to resume work.

No accurate diagnosis made until at autopsy. It is well to say, however, this man had suffered some from heat stroke a year before, which was followed by apparent epileptic convulsions. This indicated a vulnerable center in the brain.

*A Perplexing Question in Traumatic Cases.*—Did patient become unconscious and fall, producing the wound, or did the wound cause the unconsciousness? The physician must decide independently of the witnesses present, although a previous history of the case may corroborate your opinion.

*The Difficulties.*—The location and size of blood clot. Is there disease of arteries, heart, or kidneys? Is the case complicated by opium or alcoholic narcosis? Cerebral apoplexy is common in acute alcoholism after fifty years of age.

Pearce Bailey in his book on Accident and Injury, says in some fatal cases of traumatic cerebral hemorrhage mental symptoms exist without any focal sign.

If then patient is unconscious how are you to make a diagnosis?

*Differential Diagnosis in the Following Similar Conditions.*—Where there is deep coma with complete relaxation; hemorrhage into the ventricles or pons; opium and alcoholic narcosis, and uremia. It is difficult because hemiplegia cannot be ascertained; because albumin may be found in urine of each; because convulsions are not infrequent in each; because all special senses are paralysed; because the pupils are often contracted.

*What can be done toward definite diagnosis.*—Examine head for injury, examine heart and condition of arteries, examine urine for sugar and albumin, examine reflexes, examine presence or absence of rigidity or relaxation of either side and take the temperature, which is usually low in hemorrhage. Get history of case and don't give out your diagnosis for twenty-four hours.

Differential diagnosis between intracranial hemorrhage and alcoholism is not easy and mistakes are common. The physician censures the police when a man is kept in station house all night as drunk when he has cerebral hemorrhage, or fractured skull. The older physician criticises the younger men of the profession when a patient is turned away from the hospital as intoxicated, when he has cerebral hemorrhage. The people blame the profession generally for their lack of skill in these cases.

The whole trouble is in a snap diagnosis. The physician should remember that deep coma from cerebral lesion associated with alcoholism cannot always be differentially diagnosticated on the spot. That physicians who attempts it will now and then make serious mistakes. The entire difficulty could be removed by having a detention hospital, or a hospital which has an isolated room to receive such cases. The police tell us the hospitals would be kept busy if they should send all cases of unconsciousness to the hospital and the hospital authorities claim that they would continually have a nuisance in their wards. These statements are exaggerated. There are not very many patients found on the street in deep coma and who need hospital care. A hospital should always receive these cases, but they need not be kept any longer than to have a positive diagnosis made.

Why should a hospital keep delirium tremens patients who endanger the lives of those sick from disease or operation and yet turn away with an uncertain diagnosis one brought to the hospital for admission in an unconscious condition. If any should go to jail the delirium tremens patient should be moved first. Why should not a hospital have a proper room padded and isolated for those suffering from delirium tremens when the disease is so common and serious?

The hospital is the place for these cases when isolated, but never should they be put in the ward with others or where they may disturb other cases. It is too frequently done. Hospitals should meet these demands.

239 Delaware Avenue.

---

TO KEEP COCAINE.

℞ Cocaine hydrochl. . . . . . . . . . . . . . . . . . . gr. iv.

Acidi salicylici . . . . . . . . . . . . . . . . . . . . gr. ss.

Aq. dest. . . . . . . . . . . . . . . . . . . . . . ℥iij.

—*Jour. de Med. de Paris.*

